# Hepatic Ischemia and Reperfusion Injury in the Absence of Myeloid Cell-Derived COX-2 in Mice

**DOI:** 10.1371/journal.pone.0096913

**Published:** 2014-05-12

**Authors:** Sergio Duarte, Hiroyuki Kato, Naohisa Kuriyama, Kathryn Suko, Tomo-o Ishikawa, Ronald W. Busuttil, Harvey R. Herschman, Ana J. Coito

**Affiliations:** 1 Department of Surgery, The Dumont-UCLA Transplant Center, David Geffen School of Medicine at UCLA, Los Angeles, California, United States; 2 Present address: Department of Hepatobiliary Pancreatic Surgery, Mie University Graduate School of Medicine, Mie, Japan; 3 Present address: Mouse Group, Genomics Business Department, Transgenic Inc., Kobe, Japan; 4 Department of Molecular and Medical Pharmacology, Jonsson Comprehensive Cancer Center, David Geffen School of Medicine at UCLA, Los Angeles, California, United States; Universidade de Sao Paulo, Brazil

## Abstract

Cyclooxygenase-2 (COX-2) is a mediator of hepatic ischemia and reperfusion injury (IRI). While both global COX-2 deletion and pharmacologic COX-2 inhibition ameliorate liver IRI, the clinical use of COX-2 inhibitors has been linked to increased risks of heart attack and stroke. Therefore, a better understanding of the role of COX-2 in different cell types may lead to improved therapeutic strategies for hepatic IRI. Macrophages of myeloid origin are currently considered to be important sources of the COX-2 in damaged livers. Here, we used a *Cox-2^flox^* conditional knockout mouse (COX-2^−M/−M^) to examine the function of COX-2 expression in myeloid cells during liver IRI. COX-2^−M/−M^ mice and their WT control littermates were subjected to partial liver ischemia followed by reperfusion. COX-2^−M/−M^ macrophages did not express COX-2 upon lipopolysaccharide stimulation and COX-2^−M/−M^ livers showed reduced levels of COX-2 protein post-IRI. Nevertheless, selective deletion of myeloid cell-derived COX-2 failed to ameliorate liver IRI; serum transaminases and histology were comparable in both COX-2^−M/−M^ and WT mice. COX-2^−M/−M^ livers, like WT livers, developed extensive necrosis, vascular congestion, leukocyte infiltration and matrix metalloproteinase-9 (MMP-9) expression post-reperfusion. In addition, myeloid COX-2 deletion led to a transient increase in IL-6 levels after hepatic reperfusion, when compared to controls. Administration of celecoxib, a selective COX-2 inhibitor, resulted in significantly improved liver function and histology in both COX-2^−M/−M^ and WT mice post-reperfusion, providing evidence that COX-2-mediated liver IRI is caused by COX-2 derived from a source(s) other than myeloid cells. In conclusion, these results support the view that myeloid COX-2, including myeloid-macrophage COX-2, is not responsible for the hepatic IRI phenotype.

## Introduction

Liver ischemia and reperfusion injury (IRI) remains a major clinical problem in orthotopic liver transplantation (OLT) [1]. Despite improvements in surgical techniques and perioperative care, IRI causes up to 10% of early transplant failures and can significantly increase the incidence of both acute and chronic rejection [2]. Moreover, the shortage of donor organs has led to an increased use of marginal livers, which are more susceptible to IRI [3]. Hepatocellular damage caused by the IR-insult is the result of complex molecular interactions between various inflammatory mediators. A better understanding of the molecular pathophysiology of IRI should lead to improved therapeutic strategies and, thereby, to increased numbers of patients that successfully undergo liver transplantation [3].

Cyclooxygenase-2 (COX-2; prostaglandin H2 synthase), the inducible COX enzyme isoform, is among the most prominent inflammatory mediators in both acute and chronic pathological conditions [4]. COX-2 catalyzes the conversion of arachidonic acid to prostanoids (prostacyclin, prostaglandins, and thromboxanes) [5]. Several COX-2 selective non-steroidal anti-inflammatory drugs (NSAIDs) have been developed and approved for clinical use [6]. However, while COX-2 selective inhibitors have potent anti-inflammatory roles in a variety of pathological conditions [5], clinical studies have also linked their use to an increased risk of developing cardiovascular events [6], [7]. The sources of the COX-2 activity whose inhibition leads to cardiovascular risk have been postulated to be in the thymus, gut, brain [8], as well as in the vasculature [9]. COX-2 inhibition may result in decreased production of endothelial nitric oxide synthase (eNOS)-derived NO and suppression of prostacyclin (PGI(2)), a prostaglandin that is both a potent inhibitor of platelet aggregation and a powerful vasodilator [6], [9].

COX-2 expression is markedly upregulated in damaged livers after transplantation [10], and there is extensive evidence that the use of COX-2 selective inhibitors results in major beneficial effects in a number of hepatic IRI experimental models [11]-[15]. In orthogonal experiments, we showed that global COX-2 gene deletion reduces matrix metalloproteinase-9 activity, impairs neutrophil infiltration and favors a Th2-type immune response in liver IRI [14]. Considering the risks of cardiovascular events associated with the use of COX-2 inhibitors, together with the increasing appreciation that the net effects of COX-2 inhibition depend on the type of cells involved [6], [16], determining the role of distinct cell sources of COX-2 in the progression of tissue damage may provide important insights into the mechanisms of liver IRI. COX-2 expression has been largely linked to cells of the immune system, particularly to macrophages [17]. Therefore, in the present work, we used a myeloid cell-specific COX-2^−/−^ (COX-2^−M/−M^) mouse to determine the role of COX-2 expression by myeloid cells, including macrophages, in hepatic IRI.

## Methods

### Mice and model of hepatic I/R injury

**Figure 1:**
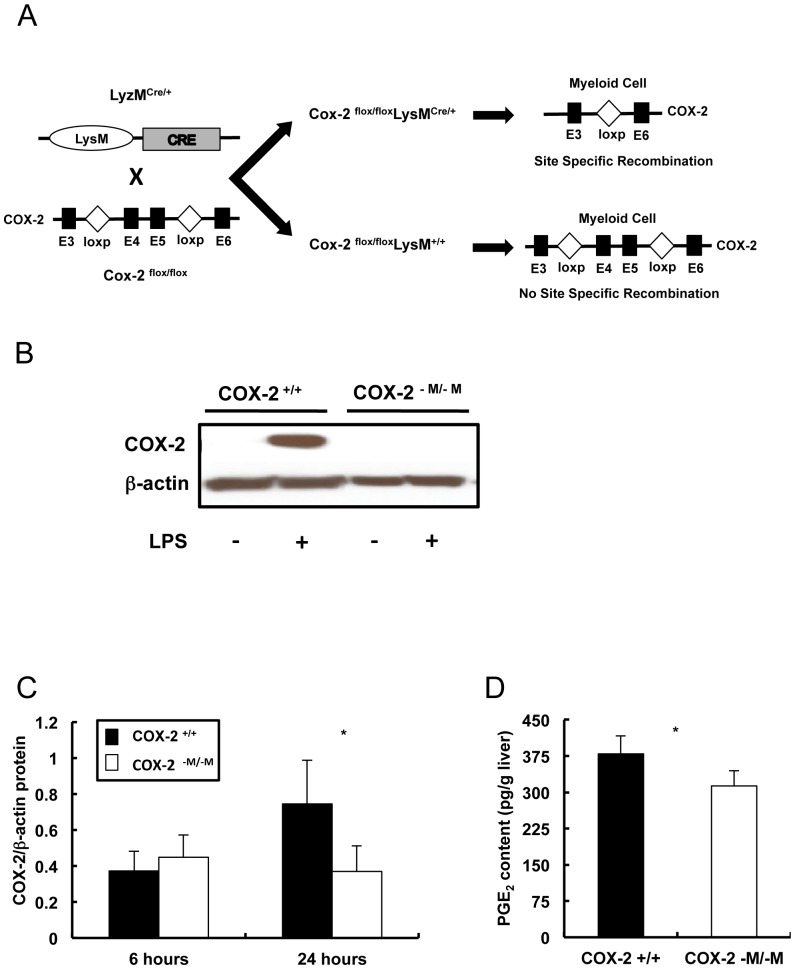
Cre-mediated, myeloid cell-specific inactivation of the COX-2 gene. COX-2 floxed mice, in which exons 4 and 5 of the COX-2 gene are flanked by loxP sites, were crossed to LysM Cre mice to obtain COX-2^−M/−M^ (COX-2flox/flox, LysMCre/+) mice and wild-type (COX-2flox/flox, LysM+/+) littermates. Myeloid cell-specific expression of cre recombinase in COX-2^−M/−M^ mice promotes cre-mediated site-specific recombination of the loxP sites, resulting in deletion of exons 4 and 5 and inactivation of COX-2 gene expression in myeloid cells (panel A). Peritoneal macrophages from COX-2^−M/−M^ mice failed to express COX-2 protein upon LPS stimulation, in contrast to COX-2^+/+^ WT macrophages, which expressed elevated levels of COX-2 protein in response to LPS (panel B). COX-2 protein expression (panel C) and PGE_2_ levels (panel D) were significantly reduced in COX-2^−M/−M^ livers at 24 h post-reperfusion, when compared with respective WT controls (n = 5–6/group; * p<0.05).

COX-2 floxed mice (*Cox-2^flox/flox^*), in which Cox-2 exons 4 and 5 are flanked by loxP sites, have been previously described [18]. LysMCre knock-in mice (B6.129P2-Lyzstm1(cre)lfo/J) were purchased from Jackson Laboratory (Bar Harbor, MA). COX-2 floxed mice, in which exons 4 and 5 of the COX-2 gene are flanked by loxP sites, were crossed to LysM Cre mice to obtain COX-2^−M/−M^ (COX-2flox/flox, LysMCre/+) mice and wild-type (COX-2flox/flox, LysM+/+) littermates; myeloid cell-specific expression of cre recombinase in COX-2^−M/−M^ mice promotes cre-mediated site-specific recombination of the loxP sites, resulting in deletion of exons 4 and 5 and inactivation of COX-2 gene expression in myeloid cells (Fig 1A). Hepatic IRI was performed as previously described [14]. Briefly, arterial and portal venous blood supplies were interrupted to the cephalad lobes of the liver for 90 minutes using an atraumatic clip and mice were sacrificed after reperfusion. The animal studies were carried out with the approval of the UCLA Animal Research Committee and followed the guidelines outlined in the "Guide for the Care and Use of Laboratory Animals" prepared by the National Academy of Sciences and published by the National Institutes of Health.

### Celecoxib administration

Celecoxib (100 mg/kg; LKT Laboratories) was administered orally by gavage to COX-2^−M/−M^ mice and COX-2^+/+^ WT control littermates 30 min before ischemia, as previously described [14]. Separate groups of COX-2^−M/−M^ and COX-2^+/+^ WT mice were similarly treated with vehicle. Celecoxib or vehicle administration had no effect on liver function in naive animals.

### Assessment of liver damage

Serum alanine transaminase (ALT) and serum aspartate transaminase (AST) levels were measured using a commercially available kit (Teco Diagnostics, Anaheim, CA), following the manufacturer's instructions. Liver specimens were fixed with a 10% buffered formalin solution, embedded in paraffin, and processed for hematoxylin and eosin (H&E) staining.

### Myeloperoxidase (MPO) assay

MPO activity was evaluated in frozen tissue homogenized in an iced solution of 0.5% hexadecyltrimethyl-ammonium and 50 mmol/L of potassium phosphate buffer solution, as described [14]. After centrifugation the supernatants were mixed in a solution of hydrogen peroxide-sodium acetate and tetramethyl benzidine (Sigma). The quantity of enzyme degrading 1 µmol/L peroxide per minute at 25°C per gram of tissue was defined as 1 unit (U) of MPO activity.

### Immunohistochemistry

Liver specimens were embedded in Tissue Tec OCT compound (Miles, Elkhart, IN) and snap frozen in liquid nitrogen and immunostaining performed on cryostat sections (0.5 µm) as described previously [19]. Appropriate rat primary antibodies against mouse macrophage antigen-1 (Mac-1; M1/70), CD68 (FA-11, Serotec), Ly-6G (1A8) (BD Biosciences, San Diego, CA), MMP-9 (AF909; R&D Systems, Minneapolis, MN) were used at optimal dilutions. Primary antibody was replaced with dilution buffer for negative controls. Bound primary antibodies were detected using biotinylated anti-rat IgG and streptavidin peroxidase–conjugated complexes (Vector Laboratories, Burlingame, CA). Sections from inflammatory tissues known to be positive for each stain were included as positive controls. Sections were evaluated blindly by counting 30 high-power fields per section.

### Esterase activity

Esterase activity of cells of granulocytic lineage was determined by a Naphthol AS-D Chloroacetate (Specific Esterase) Kit (Sigma), according to the manufacturer's instructions. Briefly, liver cryostat sections were fixed in citrate-acetone-formaldehyde solution for 30 seconds prior to incubation at 37°C in naphthol AS-D chloroacetate solution. Slides were then rinsed and mounted for analysis under the microscope.

### RT-PCR and quantitative PCR

RNA was extracted from livers with Trizol (Life Technologies) as described [14]. Reverse transcription was performed using 5 µg of total RNA in a first-strand cDNA synthesis reaction with SuperScript III RNaseH Reverse Transcriptase (LifeTechnologies), as recommended by the manufacturer. The PCR products were amplified by PCR using primers specific for each target cDNA (Table 1). The real-time PCR products were amplified in duplicates with SsoAdvanced Universal SYBR Green Supermix (Biorad, Hercules, CA). Image J software (NIH) was used for densitometry analysis.

**Table 1:** Primer Sequences.

Target Gene	Sequence
COX-2	Forward: 5′-CCAGCACTTCACCCATCAGTT-3′ (RT)
	Reverse: 5′-ACCCAGGTCCTCGCTTATGA-3′ (RT)
	Forward: 5′-CCAGATGCTATCTTTGGGGAGAC-3′
	Reverse: 5′-GCTTGCATTGATGGTGGCTG-3′
TNF-α	Forward: 5'-GGCAGGTCTACTTTGGAG-3′
	Reverse: 5'-ACATTCGAGGCTCCAGTG-3'′
IL-6	Forward: 5'-CATCCAGTTGCCTTCTTGGGA-3'
	Reverse: 5'-CATTGGGAAATTGGGGTAGGAAG-3'
IFN-γ	Forward: 5'-TACTGCCACGGCACAGTCATTGAA-3'
	Reverse: 5'-GCAGCGACTCCTTTTCCGCTTCCT-3'
IL-10	Forward: 5'-ATGCAGGACTTTAAGGGTT-3'
	Reverse: 5'-ATTTCGGAGAGAGGTACA-3'
IL-2	Forward: 5'-CTTCAAGCTCCACTTCAAGCT-3'
	Reverse: 5'-CCATCTCCTCAGAAAGTCCAC-3'
MMP-9	Forward: 5'-AGTTTGGTGTCGCGGAGCAC-3'
	Reverse: 5'-TACATGAGCGCTTCCGGCAC-3'
MCP-1	Forward: 5'-GCATGAGGTGGTTGTGAAAAA-3'
	Reverse: 5'-CTCACCTGCTGCTACTCATTC-3'
CXCL-1	Forward: 5'-TGAGCTGCGCTGTCAGTGCCT-3'
	Reverse: 5'-AGAAGCCAGCGTTCACCAGA-3'
CXCL-2	Forward: 5'-GCTGGCCACCAACCACCAGG-3'
	Reverse: 5'-AGCGAGGCACATCAGGTACG-3'
β-actin	Forward: 5′-GTGGGGCGCCCCAGGCACCA-3′
	Reverse: 5′-CTCCTTAATGTCACGCACGATTTC-3′
18S rRNA	Forward: 5′-AGAAACGGCTACCACATCCAA-3′
	Reverse: 5′-GGGTCGGGAGTGGGTAATTT-3′

### Prostaglandin E_2_ production

Concentrations of PGE2 in liver extracts were determined using a commercial enzyme immunoassay kit (Cayman Chemical) according to the manufacturer's instructions.

### Western blot analysis

Western blots were performed as described [20]. Proteins (40 µg/sample) in sodium dodecyl sulfate (SDS)-loading buffer were electrophoresed through 10–12% SDS-polyacrylamide gel electrophoresis (PAGE) and transferred to PVDF membranes. Equal protein loading was controlled by staining the gels with Coomassie brilliant blue R-250 (Bio-Rad, Hercules, CA). The membranes were incubated with a specific anti-murine COX-2 antibody (Cayman Chemical, Ann Arbor, MI). After development, membranes were stripped and reblotted with anti-actin antibody (Abcam, Cambridge, MA) for normalization. Detection on x-ray film was performed with the SuperSignal West Pico chemiluminescent substrate (Pierce Chemical, Rockford, IL) and relative quantities of protein were determined using densitometry software Image J (NIH).

### ELISA assay

Cytokine concentrations in liver extracts (40 µg of total protein) and in cell culture supernatants were measured by a sandwich ELISA (eBioscience) assay according to the manufacturer's instructions [14]. The conversion of tetramethylbenzidine by HRP was detected by measuring the absorbance at 450 nm using an ELISA plate reader (BioTek Instruments). Mouse rIL-6 and rIFN-γ from their respective ELISA kits were used as standards. Final cytokine levels were expressed as pg/ml of serum or pg/ml of culture supernatant.

### Cell isolation and culture

Isolation of hepatocytes, sinusoidal endothelial cells (SEC), and macrophages from COX-2^+/+^ and COX-2^−M/−M^ mice was performed according to previously published methods [21]–[23]. Briefly, to isolate primary murine hepatocytes and SECs, anesthetized mice were subject to a midline laparotomy and cannulation of the inferior vena cava (IVC) followed by liver perfusion with an EDTA-chelating perfusion buffer (10 mM Hepes, 0.15 M NaCl, 0.42 g/L KCl, 0.99 g/L Glucose, 2.1 g/L NaHCO3, 0.19 g/L EDTA). After perfusion with collagenase buffer (50 mM Tris–HCl, pH 7.5, 150 mM NaCl, 5 mM CaCl2 and 0.02% Brij-35), livers were minced and cells dispersed in culture medium; hepatocyte and nonparenchymal cells were separated by low-speed centrifugation methods. SECs were then purified using a two-step Percoll gradient (25/50%) and selective adherence. Macrophages were isolated 72 hours after injection of 3% thioglycollate medium into the peritoneal cavity of COX-2^+/+^ and COX-2^−M/−M^ mice. Isolated cells were cultured overnight in serum free culture medium at 5×10^5^ cells/well prior to 6 hours or 24 hours of stimulation with lipopolysaccharide (10 ng/mL, LPS, Sigma). Controls remained untreated. After cell lysis, mRNA or protein was extracted to evaluate COX-2 expression. Cytokines were measured in 6 h and 24 h cell supernatants.

### Data analysis

Results are expressed as means ± SEM. Two-group comparisons were analyzed by the two-tailed student's t-test for independent samples. Probability values of less than 0.05 were considered statistically significant.

## Results

### COX-2 protein expression was reduced in COX-2^−M/−M^ mouse livers after liver IRI

COX-2 expression is inducible in human and murine macrophages by LPS [24]. In our settings, LPS-stimulated COX-2^+/+^ WT macrophages expressed high levels of COX-2 protein, while LPS-stimulated COX-2^–M/-M^ macrophages failed to express this pro-inflammatory mediator (Fig. 1B), confirming that the COX-2 gene was effectively deleted in the macrophages of COX-2^−M/−M^ mice [25]. In livers, COX-2 protein expression was virtually undetectable (data not shown) in COX-2^−M/−M^ and COX-2^+/+^ mice prior to surgery (naïve livers), but it was readily observable after reperfusion. However, COX-2 protein levels were significantly reduced in COX-2^−M/−M^ livers at 24 h post-IRI when compared to respective COX-2^+/+^ WT controls (Fig. 1C). In addition, PGE_2_ levels were reduced by approximately 20% (p<0.05) in COX-2^−M/−M^ livers after 24 of reperfusion (Fig. 1D).

### Absence of myeloid COX-2 did not protect against I/R-induced liver damage

**Figure 2:**
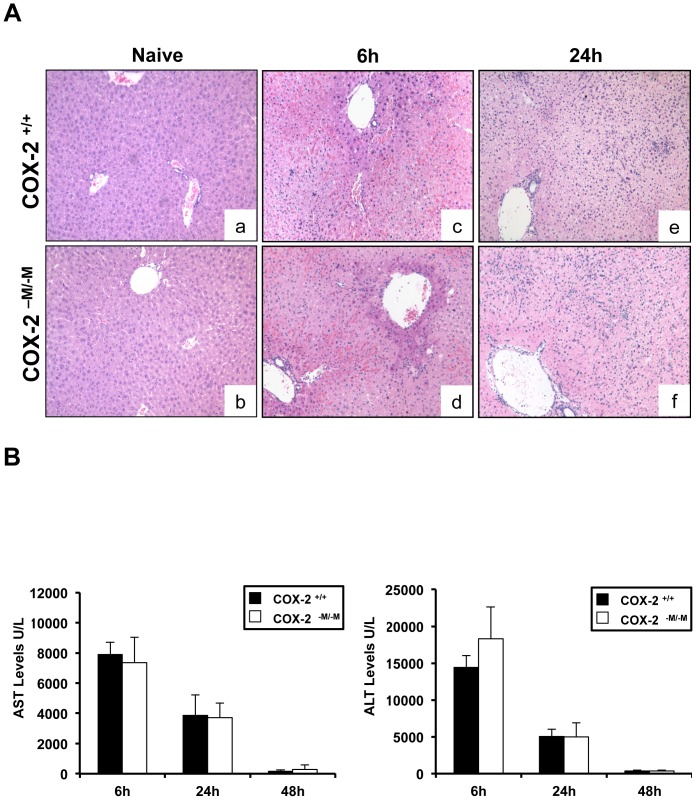
Histology and serum transaminase levels in COX-2^−M/−M^ and COX-2^+/+^ mice. H&E staining of liver sections (panel A) from COX-2^+/+^ mice (a, c, e) and COX-2^-M/-M^ mice (b, d, f) revealed good histological preservation in naïve livers (a, b) and comparable extensive hepatic necrosis, vascular congestion, and disruption of liver architecture in COX-2^+/+^ livers (c, e) and COX-2^−M/−M^ livers (d, f) at 6h (c, d), and 24h (e, f) after IRI. Serum AST and ALT levels (panel B) were also similar in COX-2^+/+^ mice (black bars) and COX-2^−M/−M^ mice (white bars) after IRI; (n = 5-6/group).

We previously demonstrated that global COX-2 gene deletion ameliorates hepatic IRI [14]. Therefore, to determine the significance of myeloid cell-derived COX-2, both *COX-2^−M/−M^* and their littermate *COX-2^+/+^* mice were subject to this well-established model of partial liver IRI. Livers from both *COX-2^−M/−M^* and *COX-2^+/+^* mice developed similar levels of injury, characterized by significant vascular congestion, lobular architecture disruption, and extensive necrosis after reperfusion (Fig. 2A). The serum transaminase levels (U/L) were comparable in *COX-2^−M/−M^* and *COX-2^+/+^* mice at 6 h (AST: 7,349±1,686 vs. 7,910±815; sALT: 18,281±4,371 vs. 14,424±1,608), 24 h (AST: 3,707±982 vs. 3,853±1,368; ALT: 5,000±1,909 vs. 5,053±989) and 48 h (AST: 279±293 vs. 156±95; ALT: 380±93 vs. 357±109) hours post-IRI (Fig. 2B).

### Leukocyte accumulation and MMP-9 expression were comparable in COX-2^−M/−M^ and COX-2^+/+^ livers after IRI

**Figure 3:**
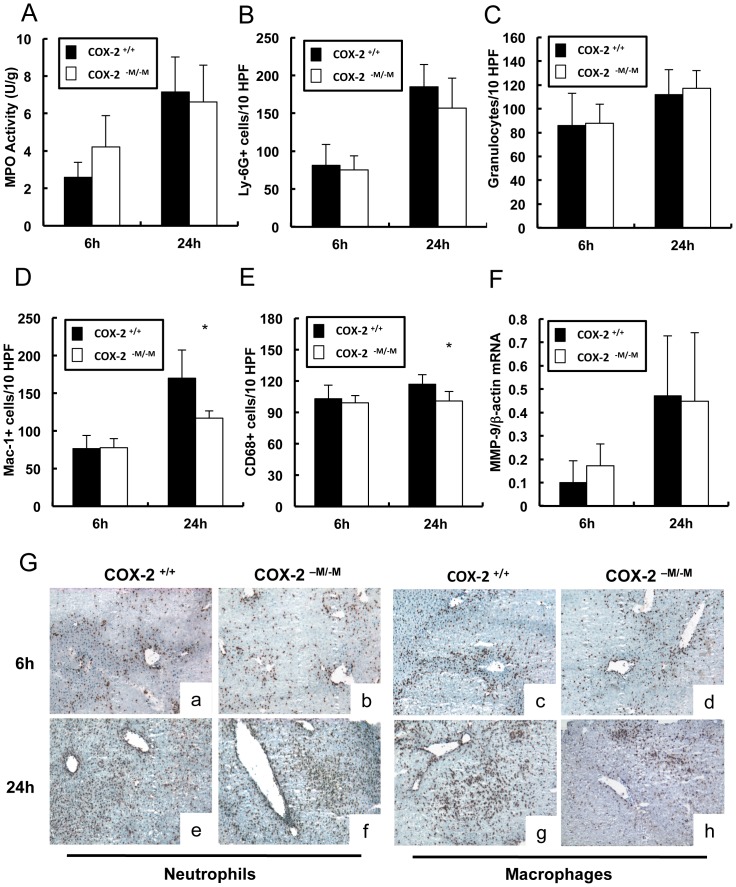
Leukocyte infiltration in COX-2^−M/−M^ and COX-2^+/+^ mice. MPO enzymatic activity (panel A) was statistically similar in COX-2^−M/−M^ and COX-2^+/+^ livers at 6 h and 24 h post-IRI. Ly-6G^+^ neutrophil (panel B) and granulocyte (panel C) infiltration were also comparable in COX-2^−M/−M^ and COX-2^+/+^ livers after IRI. Mac-1^+^ (panel D) and CD68 (panel E) infiltrating macrophages were significantly reduced in COX-2^−M/−M^ livers at 24 h post-reperfusion, but were statistically indistinguishable in COX-2^−M/−M^ and COX-2^+/+^ livers at 6 h after IRI. No statistical differences in MMP-9 expression (panel F) could be demonstrated in livers of COX-2^−M/−M^ and COX-2^+/+^ mice post-IRI. Representative immunostaining (panel G) of infiltrating Ly-6G^+^ (a,b,e,f) and Mac-1^+^ (c,d,g,h) leukocytes in livers of COX-2^+/+^ (a,c,e,g) and COX-2^−M/−M^ (b,d,f,h) mice at 6 h (a to d) and 24 h (e to h) post IRI; (n = 5–6/group; * indicates p<0.05).

We next tested whether absence of myeloid COX-2 affects leukocyte recruitment in hepatic IRI. *COX-2^−M/−M^* and *COX-2^+/+^* livers had similar levels of myeloperoxidase (MPO) activity (U/g) at 6 h (4.2±0.8 vs. 2.78±1.7) and 24 h (6.6±2.0 vs. 7.2±1.9) post-IRI (Fig. 3A). Moreover, *COX-2^−M/−M^* and *COX-2^+/+^* livers showed almost similar levels of Ly-6G^+^ neutrophil (6 h: 75±18 vs. 81±28; 24 h: 157±39 vs. 185±30), granulocyte (6 h: 88±16 vs. 86±27; 24 h: 117±15 vs. 112±21), Mac-1^+^ macrophage (6 h: 78±12 vs. 76±18; 24 h: 117±15 vs. 170±38, p<0.05; 48 h: 161±30 vs. 160±21), and CD68 leukocyte (6 h: 99±7 vs. 103±13; 24 h: 101±9 vs. 117±9, p<0.05) infiltration after reperfusion (Fig. 3B, C, D, E, and G). While we observed a statistically significant difference in Mac-1/CD68 macrophage infiltration in COX-2^−M/−M^ mice at 24 h after liver IRI, the COX-2^−M/−M^ livers were still highly infiltrated and it seems unlikely that a modest reduction in these macrophage cell counts would greatly affect the progression of liver IRI in these mice. The expression of MMP-9, an important mediator of leukocyte infiltration in damaged livers, was also not affected by the absence of myeloid COX-2; COX-2^−M/−M^ and COX-2^+/+^ livers expressed similar levels of MMP-9 mRNA (6 h: 0.17±0.09 vs. 0.10±0.09; 24 h: 0.45±0.29 vs. 0.47±0.26) after reperfusion (Fig. 3F).

### Absence of myeloid COX-2 resulted in an early increase of IL-6 levels after hepatic IRI

**Figure 4:**
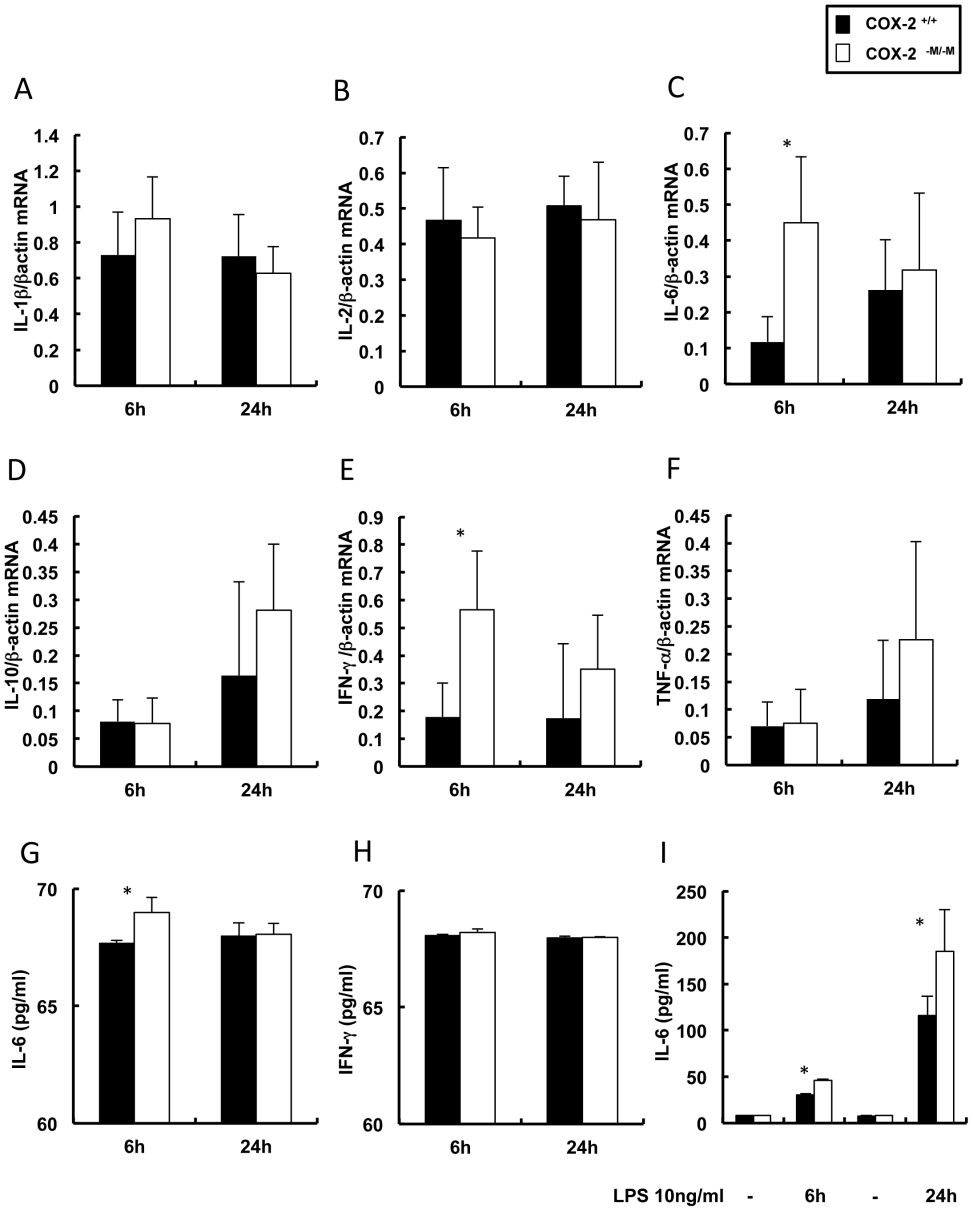
Cytokine expression in COX-2^−M/−M^ and COX-2^+/+^ mice. Levels of IL-1β (panel A), IL-2 (panel B), IL-10 (panel D) and TNF-α (panel F) mRNA expression were similar in livers of COX-2^+/+^ and COX-2^−M/−M^ mice at 6 h and 24 h post-IRI. In contrast, depletion of myeloid cell specific COX-2 resulted in a significantly increased IL-6 (panel C) and IFN-γ (panel E) mRNA expression at 6 h post-IRI, compared to respective controls; however, both COX-2^−M/−M^ and COX-2^+/+^ livers expressed comparable levels of L-6 and IFN-γ at 24 h post-reperfusion. Serum IL-6 protein levels (panel G) were significantly increased in COX-2^−M/−M^ mice at 6 h post-reperfusion, whereas changes in serum IFN-γ protein (panel H) didn't reach statistical significance. IL-6 protein levels were markedly increased in cultured COX-2^−M/−M^ macrophages after 6 h and 24 h of LPS stimulation, compared with respective control COX-2^+/+^ stimulated macrophages (panel I); (n = 6/group; in vitro data are expressed as means ± SD of four independent experiments; * indicates p<0.05).

**Figure 5:**
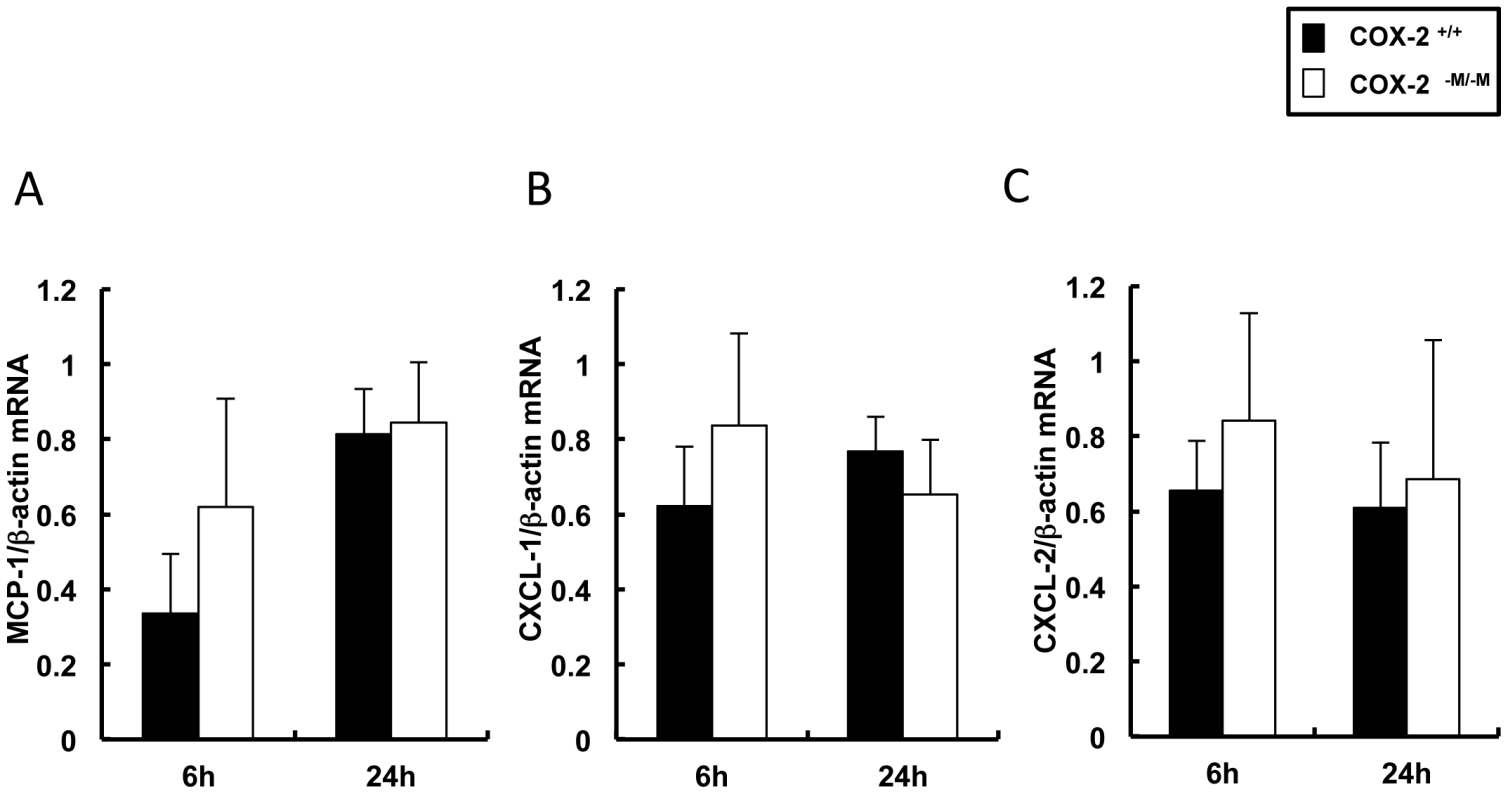
Chemokine expression in livers of COX-2^−M/−M^ and COX-2^+/+^ mice. The levels of the chemokines MCP-1 (panel A), CXCL-1 (panel B) and CXCL-2 (panel C) were statistically indistinguishable in the livers of COX-2^−M/−M^ mice and COX-2^+/+^ mice at 6 h and 24 h after IRI, (n = 6/group).

COX-2 inhibition plays a prominent role in regulating Th1 and Th2 type responses, suppressing IL-2 expression and increasing IL-10 levels in several pathological conditions [26], [27], which include hepatic IRI [14]. In the present setting, absence of myeloid COX-2 did not notably affect the mRNA expressions of IL-1β (6 h: 0.93±0.23 vs. 0.73±0.24; 24 h: 0.63±0.15 vs. 0.72±0.23), IL-2 (6 h: 0.42±0.09 vs. 0.47±0.15; 0.47±0.16 vs. 0.51±0.08), IL-10 (6 h: 0.08±0.05 vs. 0.08±0.04; 24 h: 0.28±0.12 vs. 0.16±0.17), and TNF-α (6 h: 0.08±0.06 vs. 0.07±0.04; 24 h: 0.23±0.18 vs. 0.12±0.11) post-IRI (Fig. 4A, B, D, and F). In contrast, the mRNA levels of IL-6 (0.45±0.18 vs. 0.12±0.07; p<0.05) and IFN-γ (0.57±0.21 vs. 0.18±0.12; p<0.05) were significantly upregulated in *COX-2^−M/−M^* livers 6 h after reperfusion IRI (Fig. 4C and E). Moreover, IL-6 protein levels were significantly increased in the serum of *COX-2^−M/−M^* mice at 6 h post-IRI (Fig. 4G). Differences in serum IFN-γ protein in *COX-2^−M/−M^* mice and COX-2^+/+^ mice did not reach statistical significance at 6 h post-reperfusion (Fig. 4H). Cytokine upregulation was transient, as both *COX-2^−M/−M^* and *COX-2^+/+^* mice showed comparable liver mRNA expression (IL-6: 0.32±0.21 vs. 0.26±0.14; IFN-γ: 0.35±0.19 vs. 0.17±0.27) and serum protein levels after 24 hours of IRI, (Fig 4C, E, G, and H). To investigate whether COX-2 expression in macrophages affects cytokine expression, we measured IL-6 and IFN-γ production in cultured *COX-2^−M/−M^* and COX-2^+/+^ macrophages with or without LPS stimulation. Indeed, IL-6 expression was significantly upregulated in *COX-2^−M/−M^* macrophages after 6 and 24 hours of LPS stimulation, compared to LPS-stimulated COX-2^+/+^ macrophages, (Fig. 4I). IFN-γ expression was virtually undetectable in the cultured macrophages before and after LPS stimulation. Additionally, lack of myeloid COX-2 did not affect the liver expression of the macrophage chemoattractant MCP-1 (6 h: 0.61±0.29 vs. 0.34±0.16; 24 h: 0.84±0.16 vs. 0.81±0.12), and of the neutrophil chemoattractants KC/CXCL-1 (6 h: 0.84±0.25 vs. 0.62±0.16; 24 h: 0.65±0.15 vs. 0.77±0.09) and MIP-2/CXCL-2 (6 h: 0.84±0.29 vs. 0.66±0.13; 24 h: 0.69±0.37 vs. 0.61±0.17) when compared to respective controls after IRI (Fig. 5).

### Hepatocytes and sinusoidal endothelial cells are potential sources of COX-2 expression in hepatic IRI

**Figure 6:**
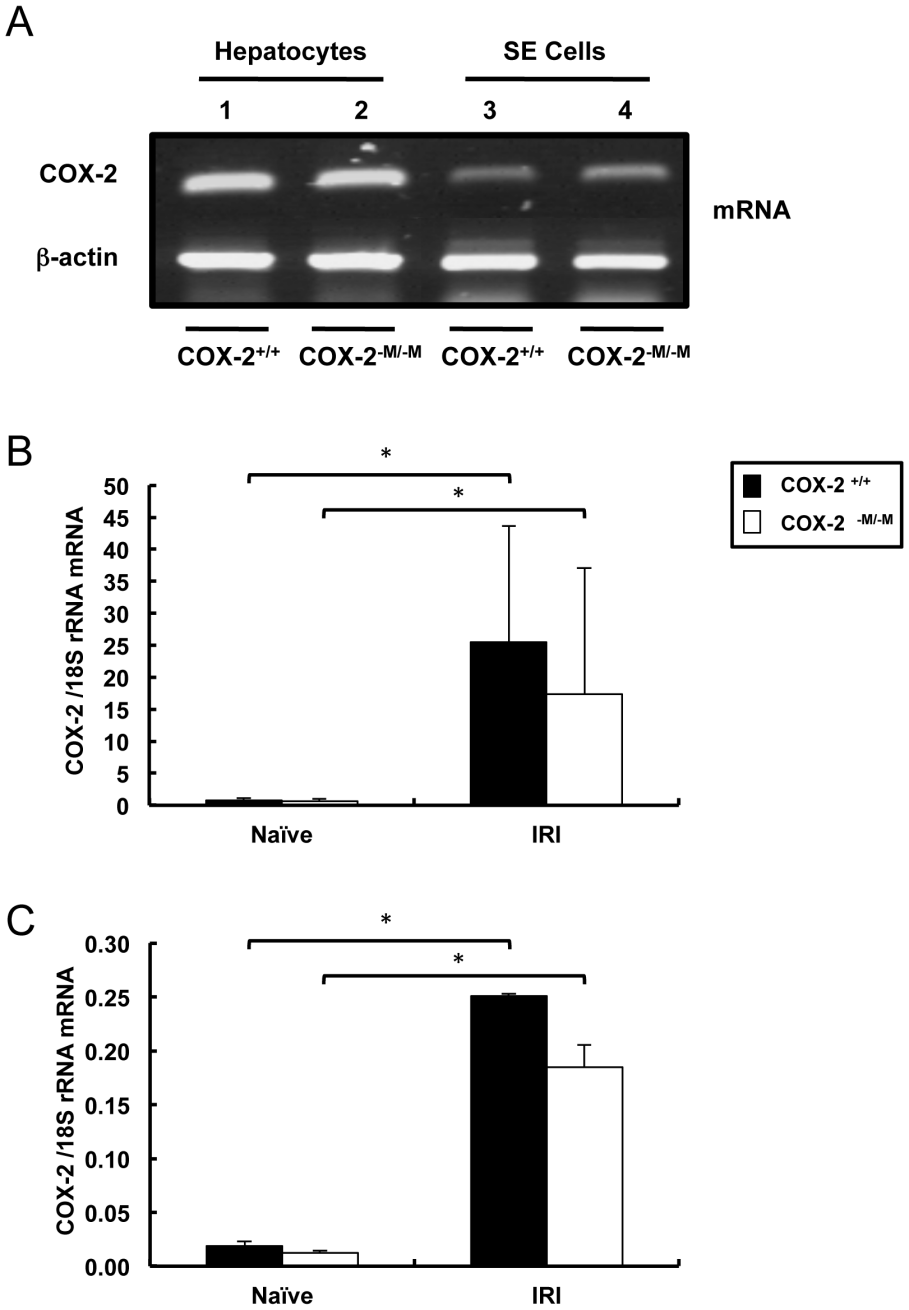
COX-2 expression in isolated hepatocytes and sinusoidal endothelial cells. RT-PCR analysis of COX-2 mRNA (panel A) in hepatocytes (lanes 1 and 2) and sinusoidal endothelial cells (lanes 3 and 4) isolated from COX-2^+/+^ (lanes 1 and 3) and COX-2^−M/−M^ (lanes 2 and 4) mice. Real-time PCR analysis of COX-2 mRNA expression in hepatocytes (panel B) and sinusoidal endothelial cells (panel C) isolated from COX-2^−M/−M^ mice and COX-2^+/+^ mice prior to surgery (naïve) and after 6 h of reperfusion; these analyses demonstrated that COX-2 mRNA was markedly upregulated in hepatocytes and sinusoidal endothelial cells isolated from both COX-2^−M/−M^ and respective wild-type controls after IRI. However, the elevated levels following reperfusion did not differ statistically between either hepatocytes or endothelial cells from COX-2^−M/−M^ versus COX-2^+/+^ mice (in vitro data is expressed as mean ± SD of three independent experiments; * indicates p<0.05).

Given the difficulty in characterizing the sources of COX-2 in mouse livers by immunohistochemical methods, we isolated hepatocytes and sinusoidal endothelial cells from both *COX-2^−M/−M^* and *COX-2^+/+^* mice. COX-2 mRNA was easily detected in the preparations of isolated cells (Fig. 6 A). Moreover, COX-2 mRNA expression was markedly increased in isolated hepatocytes and sinusoidal endothelial cells from COX-2^−M/−M^ and COX-2^+/+^ mice after liver IRI, compared to cells isolated from COX-2^−M/−M^ and COX-2^+/+^ naïve livers. However, the IRI-induced COX-2 levels in hepatocytes and sinusoidal endothelial cells from COX-2^−M/−M^ and COX-2^+/+^ mice did not differ significantly from one another; thus hepatocyte and sinusoidal endothelial cell compensation does not appear to account for the difference in macrophage-mediated COX-2 modulation of IRI damage (Fig. 6 B, and C). It is, of course, possible that isolated cells *in vitro* may not accurately mimic what has happened *in vivo*. These results provide evidence that, in addition to myeloid cells, hepatocytes and sinusoidal endothelial cells are also capable of expressing COX-2; these alternative cell types are, consequently, likely potential sources of the COX-2 necessary to mediate liver damage after IRI.

### Celecoxib administration ameliorated liver IRI in *COX-2^−M/−M^* mice

**Figure 7:**
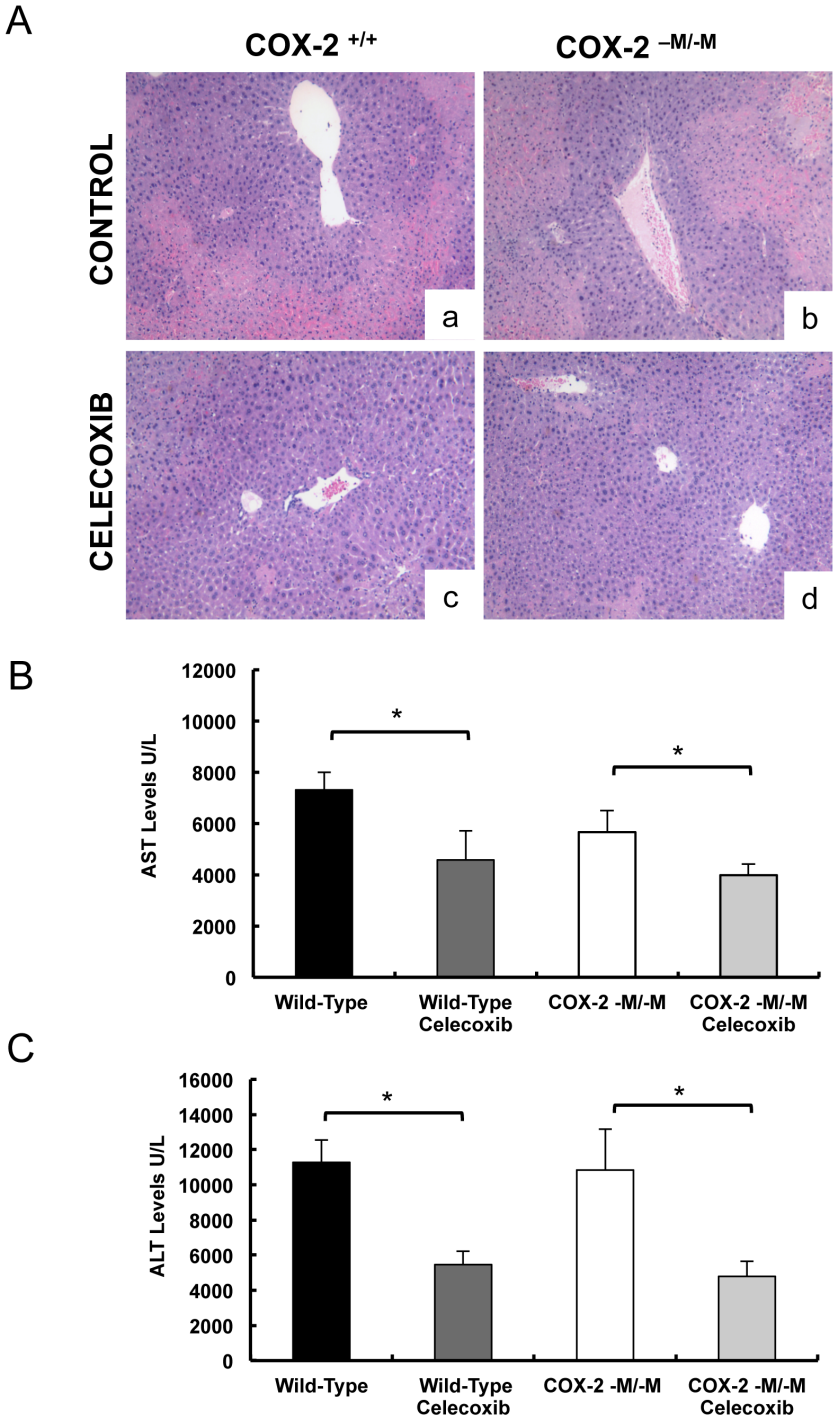
Celecoxib inhibition of hepatic IRI for both COX-2^−M/−M^ mice and COX-2^+/+^ mice. Liver H&E staining (panel A) demonstrated that administration of celecoxib, a selective COX-2 inhibitor, markedly reduced hepatocellular necrosis and vascular congestion in both COX-2^+/+^ mice (c) and COX-2^−M/−M^ mice (d), when compared with respective vehicle treated COX-2^+/+^ mice (a) and COX-2^−M/−M^ mice (b) controls 6 h post IRI. Celecoxib therapy significantly reduced the serum AST (panel B) and ALT (panel C) levels in both COX-2^+/+^ mice (dark grey bars) and COX-2^−M/−M^ mice (light grey bars), when compared to respective vehicle treated control COX-2^+/+^ mice (black bars) and COX-2^−M/−M^ mice (white bars) after 6 h of hepatic IRI (n = 5/group; * indicates p<0.05).

To further support the concept that COX-2-mediated liver IRI is caused by COX-2 derived from sources other than myeloid cells, *COX-2^−M/−M^* and *COX-2^+/+^* mice were treated with celecoxib, a selective COX-2 inhibitor, prior to surgery. Celecoxib administration significantly reduced liver damage and improved liver function, in both *COX-2^−M/−M^* and in *COX-2^+/+^* mice, after IRI. Celecoxib-treated *COX-2^−M/−M^* and *COX-2^+/+^* livers presented significantly improved histological preservation, when compared to vehicle-treated *COX-2^−M/−M^* and *COX-2^+/+^* livers, which were characterized by extensive vascular edema/congestion and necrosis (Fig. 7A). Moreover, celecoxib therapy reduced the transaminase levels (U/L) in both *COX-2^−M/−M^* (AST: 3,985±443 vs. 5,666±831, p<0.05; ALT:4,788±871 vs. 10,846±2321, p<0.05) and *COX-2^+/+^* (AST:4,579±1,143 vs. 7,315±689 p<0.05; ALT: 5,463±948 vs. 11,282±1,276, p<0.05) mice at 6 h post-reperfusion (Fig. 7B, and C). Considered together, these data clearly demonstrate that, although total pharmacological or genetic ablation of COX-2 activity improves hepatic IRI [14], selective myeloid COX-2 gene inactivation has virtually no effect in the progression of liver damage in response to IRI.

## Discussion

Observations that COX-2 is a key mediator of acute and chronic inflammation led to the development of several COX-2-selective non-steroidal anti-inflammatory drugs. Some of these COX-2-selective inhibitors, as well as total COX-2^−/−^ gene deletion, are beneficial in various hepatic experimental IRI models in mice, rats and dogs [11], [12], [14], [15], [28]. However, while COX-2 selective inhibitors seem to have significantly lower gastrointestinal complications when compared to non-selective NSAIDs, their use has been associated with higher risks of heart attack and stroke [6]. On the other hand, recent observations have suggested that the increased cardiovascular risks are due to inhibition of COX-2 in specific tissues of the body [8] rather then in the vasculature [9]. The latter data, together with growing evidence supporting the view that the net effect of COX-2 inhibition depends on the underlying disease process [16], have raised considerable interest in determining the functions of COX-2 derived from distinct cell sources in the progression of COX-2-mediated tissue injury.

COX-2 is virtually undetectable in most tissues in the absence of stimulation. However, COX-2 expression is induced in macrophages and in other leukocytes of myeloid origin during acute inflammation [29]. Moreover, macrophages have been identified as important sources of COX-2 in damaged rat livers [30]. In this study, we used a mouse model in which COX-2 has been deleted only in myeloid cells to determine the role of myeloid cell-derived COX-2, including macrophage-derived COX-2, in hepatic IRI progression. COX-2^−M/−M^ macrophages failed to express COX-2 upon LPS stimulation in vitro. In a somewhat surprising result, to us, COX-2 depletion in myeloid cells did not affect hepatic IRI. In contrast, a previous study in an air pouch model demonstrated that the absence of myeloid COX-2 significantly attenuated acute inflammation [25]. COX-2^−M/−M^ mice presented extensive hepatocellular necrosis, liver architectural damage, and severe impairment of liver function after IRI; indeed, the results with mice that had a myeloid-specific COX-2 gene deletion were essentially indistinguishable by conventional histology from those obtained with COX-2^+/+^ mice. Moreover, absence of myeloid COX-2 expression had little effect on macrophage recruitment and no impact on neutrophil infiltration. COX-2^−M/−M^ and COX-2^+/+^ livers were both heavily infiltrated by macrophages and neutrophils, and the transient decrease in macrophage infiltration observed at 24 h post-reperfusion in the COX-2^−M/−M^ mice was not sufficient to ameliorate liver IRI. Expression of MMP-9, an important mediator of leukocyte recruitment [31], as well as MCP-1, CXCL-1, and CXCL-2 chemokine levels were essentially unchanged in the absence of myeloid COX-2 in the damaged livers.

We have previously observed that total COX-2 gene deletion, in addition to ameliorating hepatic IRI, results in a Th2-type cytokine profile [14]. In contrast, targeted myeloid COX-2 gene deletion did not affect IL-2 or IL-10 expression in post-liver IRI mice, but did result in slightly increased IL-6 and IFN-γ levels. Moreover, compared to isolated COX-2^+/+^ macrophages, COX-2^−M/−M^ macrophages expressed significantly higher levels of IL-6 upon LPS stimulation. IL-6 is a hepatoprotective cytokine in physiological doses, but this cytokine inhibits liver regeneration when expressed in elevated levels [32]. These data suggest that myeloid cell-derived COX-2 may have a regulatory role on inflammatory responses, and its inhibition might be detrimental early after IRI. In this regard, using targeted myeloid cell COX-2 gene deletion, we have demonstrated that myeloid cell-derived COX-2 has a protective role in a murine colitis model [33]. Overall, our data support the view that the beneficial effects in hepatic IRI observed either by COX-2 selective inhibitors or by global COX-2 gene deletion are the result of COX-2 depletion in non-myeloid cells.

We isolated hepatocytes and sinusoidal endothelial cells from COX-2-M/-M and COX-2^+/+^ livers to evaluate their capability to express COX-2. In contrast to stimulated macrophages from COX-2^−M/−M^ mice, which failed to express COX-2, the COX-2 levels in both hepatocytes and sinusoidal endothelial cells from COX-2^−M/−M^ mice were substantially elevated following isolation from livers of mice subject to IRI. These data support the conclusion that non-myeloid cells are the potential sources of the COX-2 activity that mediates liver IR damage. However, the lack of a substantial difference in the levels of COX-2 in these cells from COX-2^−M/−M^ and COX-2^+/+^ mice following IRI suggests that neither hepatocytes neither endothelial cells are producing elevated COX-2 levels in a compensatory reaction to the absence of myeloid COX-2 during the IRI response. It is, of course, possible that isolated cells in vitro may not accurately mimic what has happened in vivo. The assumption of a different cell-type source for the COX-2 expression that mediates liver IRI is further supported by our celecoxib experiments; this COX-2 selective inhibitor was equally effective in significantly reducing liver damage and improving liver function after IRI in both targeted myeloid COX-2^−M/−M^ deficient mice and COX-2^+/+^ mice. Further experimentation will be required to identify the cell-type source(s) for the COX-2 effective in the progression of liver damage after the I/R-insult.

In summary, using mice in which COX-2 has been ablated selectively in myeloid cells, our results clearly show that myeloid COX-2, including macrophage COX-2, is not responsible for the hepatic IRI phenotype. Moreover, these results suggest that COX-2 mediated liver IRI results from COX-2 expressed predominantly early after reperfusion by sources other than myeloid cells. The use of other conditional COX-2^−/−^ mice, such as those made to deplete COX-2 from hepatocytes and/or endothelial cells, might explain the beneficial effects observed with COX-2 selective inhibitors or by global COX-2 gene deletion. Such studies could be of substantial importance in the development of COX-2 targeted therapies in hepatic IRI.

## References

[1] Howard TK, Klintmalm GB, Cofer JB, Husberg BS, Goldstein RM, et al (1990) The influence of preservation injury on rejection in the hepatic transplant recipient. Transplantation 49: 103–107. 2300999 10.1097/00007890-199001000-00023

[2] Henderson JM (1999) Liver transplantation and rejection: an overview. Hepatogastroenterology 46 Suppl 2 1482–1484. 10431709

[3] Busuttil RW, Tanaka K (2003) The utility of marginal donors in liver transplantation. Liver Transpl 9: 651–663. 12827549 10.1053/jlts.2003.50105

[4] Kim SF, Huri DA, Snyder SH (2005) Inducible nitric oxide synthase binds, S-nitrosylates, and activates cyclooxygenase-2. Science 310: 1966–1970. 16373578 10.1126/science.1119407

[5] Herschman HR, Talley JJ, DuBois R (2003) Cyclooxygenase 2 (COX-2) as a target for therapy and noninvasive imaging. Mol Imaging Biol 5: 286–303. 14630509 10.1016/j.mibio.2003.09.006

[6] Cannon CP, Cannon PJ (2012) Physiology. COX-2 inhibitors and cardiovascular risk. Science 336: 1386–1387. 22700906 10.1126/science.1224398

[7] Ray WA, Stein CM, Daugherty JR, Hall K, Arbogast PG, et al (2002) COX-2 selective non-steroidal anti-inflammatory drugs and risk of serious coronary heart disease. Lancet 360: 1071–1073. 12383990 10.1016/S0140-6736(02)11131-7

[8] Kirkby NS, Zaiss AK, Urquhart P, Jiao J, Austin PJ, et al (2013) LC-MS/MS confirms that COX-1 drives vascular prostacyclin whilst gene expression pattern reveals non-vascular sites of COX-2 expression. PLoS One 8: e69524. 23874970 10.1371/journal.pone.0069524 PMC3711559

[9] Yu Y, Ricciotti E, Scalia R, Tang SY, Grant G, et al (2012) Vascular COX-2 modulates blood pressure and thrombosis in mice. Sci Transl Med 4: 132ra154. 10.1126/scitranslmed.3003787 PMC3882087 22553252

[10] Moore C, Shen XD, Fondevila C, Gao F, Coito AJ (2005) Blockade of fibronectin-alpha4beta1 adhesive interactions down-regulates cyclooxygenase-2 inducible nitric oxide synthase and prolongs recipient survival in a 24-hour model of cold hepatic ischemia-reperfusion injury. Transplant Proc 37: 1682–1683. 15919429 10.1016/j.transproceed.2005.03.146

[11] Takeyoshi I, Sunose Y, Iwazaki S, Tsutsumi H, Aiba M, et al (2001) The effect of a selective cyclooxygenase-2 inhibitor in extended liver resection with ischemia in dogs. J Surg Res 100: 25–31. 11516201 10.1006/jsre.2001.6211

[12] Kobayashi M, Takeyoshi I, Kurabayashi M, Matsumoto K, Morishita Y (2007) The effects of a cyclooxygenase-2 inhibitor, FK3311, on total hepatic ischemia-reperfusion injury of the rat. Hepatogastroenterology 54: 522–526. 17523312

[13] Ozturk H, Gezici A (2006) The effect of celecoxib, a selective COX-2 inhibitor, on liver ischemia/reperfusion-induced oxidative stress in rats. Hepatol Res 34: 76–83. 16384742 10.1016/j.hepres.2005.11.003

[14] Hamada T, Tsuchihashi S, Avanesyan A, Duarte S, Moore C, et al (2008) Cyclooxygenase-2 deficiency enhances Th2 immune responses and impairs neutrophil recruitment in hepatic ischemia/reperfusion injury. J Immunol 180: 1843–1853. 18209082 10.4049/jimmunol.180.3.1843 PMC3589995

[15] Stoffels B, Yonezawa K, Yamamoto Y, Schafer N, Overhaus M, et al (2011) Meloxicam, a COX-2 inhibitor, ameliorates ischemia/reperfusion injury in non-heart-beating donor livers. Eur Surg Res 47: 109–117. 21757922 10.1159/000329414

[16] Salinas G, Rangasetty UC, Uretsky BF, Birnbaum Y (2007) The cycloxygenase 2 (COX-2) story: it's time to explain, not inflame. J Cardiovasc Pharmacol Ther 12: 98–111. 17562780 10.1177/1074248407301172

[17] Schwarz NT, Kalff JC, Turler A, Engel BM, Watkins SC, et al (2001) Prostanoid production via COX-2 as a causative mechanism of rodent postoperative ileus. Gastroenterology 121: 1354–1371. 11729115 10.1053/gast.2001.29605

[18] Ishikawa TO, Herschman HR (2006) Conditional knockout mouse for tissue-specific disruption of the cyclooxygenase-2 (Cox-2) gene. Genesis 44: 143–149. 16496341 10.1002/gene.20192

[19] Hamada T, Duarte S, Tsuchihashi S, Busuttil RW, Coito AJ (2009) Inducible nitric oxide synthase deficiency impairs matrix metalloproteinase-9 activity and disrupts leukocyte migration in hepatic ischemia/reperfusion injury. Am J Pathol 174: 2265–2277. 19443702 10.2353/ajpath.2009.080872 PMC2684191

[20] Duarte S, Hamada T, Kuriyama N, Busuttil RW, Coito AJ (2012) TIMP-1 deficiency leads to lethal partial hepatic ischemia and reperfusion injury. Hepatology 56: 1074–1085. 22407827 10.1002/hep.25710 PMC3386467

[21] Duarte S, Shen XD, Fondevila C, Busuttil RW, Coito AJ (2012) Fibronectin-alpha4beta1 interactions in hepatic cold ischemia and reperfusion injury: regulation of MMP-9 and MT1-MMP via the p38 MAPK pathway. Am J Transplant 12: 2689–2699. 22812390 10.1111/j.1600-6143.2012.04161.x PMC3459169

[22] Zhang Y, Castellani LW, Sinal CJ, Gonzalez FJ, Edwards PA (2004) Peroxisome proliferator-activated receptor-gamma coactivator 1alpha (PGC-1alpha) regulates triglyceride metabolism by activation of the nuclear receptor FXR. Genes Dev 18: 157–169. 14729567 10.1101/gad.1138104 PMC324422

[23] Braet F, Kalle WH, De Zanger RB, De Grooth BG, Raap AK, et al (1996) Comparative atomic force and scanning electron microscopy: an investigation on fenestrated endothelial cells in vitro. J Microsc 181: 10–17. 8627618 10.1046/j.1365-2818.1996.72348.x

[24] Giroux M, Descoteaux A (2000) Cyclooxygenase-2 expression in macrophages: modulation by protein kinase C-alpha. J Immunol 165: 3985–3991. 11034408 10.4049/jimmunol.165.7.3985

[25] Narasimha AJ, Watanabe J, Ishikawa TO, Priceman SJ, Wu L, et al (2010) Absence of myeloid COX-2 attenuates acute inflammation but does not influence development of atherosclerosis in apolipoprotein E null mice. Arterioscler Thromb Vasc Biol 30: 260–268. 19926832 10.1161/ATVBAHA.109.198762 PMC2859183

[26] Ni J, Shu YY, Zhu YN, Fu YF, Tang W, et al (2007) COX-2 inhibitors ameliorate experimental autoimmune encephalomyelitis through modulating IFN-gamma and IL-10 production by inhibiting T-bet expression. J Neuroimmunol 186: 94–103. 17442406 10.1016/j.jneuroim.2007.03.012

[27] Suzuki J, Ogawa M, Futamatsu H, Kosuge H, Tanaka H, et al (2006) A cyclooxygenase-2 inhibitor alters Th1/Th2 cytokine balance and suppresses autoimmune myocarditis in rats. J Mol Cell Cardiol 40: 688–695. 16499924 10.1016/j.yjmcc.2006.01.006

[28] Oshima K, Yabata Y, Yoshinari D, Takeyoshi I (2009) The effects of cyclooxygenase (COX)-2 inhibition on ischemia-reperfusion injury in liver transplantation. J Invest Surg 22: 239–245. 19842898 10.1080/08941930903040080

[29] McAdam BF, Catella-Lawson F, Mardini IA, Kapoor S, Lawson JA, et al (1999) Systemic biosynthesis of prostacyclin by cyclooxygenase (COX)-2: the human pharmacology of a selective inhibitor of COX-2. Proc Natl Acad Sci U S A 96: 272–277. 9874808 10.1073/pnas.96.1.272 PMC15129

[30] Wojcik M, Ramadori P, Blaschke M, Sultan S, Khan S, et al (2012) Immunodetection of cyclooxygenase-2 (COX-2) is restricted to tissue macrophages in normal rat liver and to recruited mononuclear phagocytes in liver injury and cholangiocarcinoma. Histochem Cell Biol 137: 217–233. 22131058 10.1007/s00418-011-0889-9 PMC3262142

[31] Coito AJ (2011) Leukocyte transmigration across endothelial and extracellular matrix protein barriers in liver ischemia/reperfusion injury. Curr Opin Organ Transplant 16: 34–40. 21150609 10.1097/MOT.0b013e328342542e PMC3156893

[32] Wustefeld T, Rakemann T, Kubicka S, Manns MP, Trautwein C (2000) Hyperstimulation with interleukin 6 inhibits cell cycle progression after hepatectomy in mice. Hepatology 32: 514–522. 10960443 10.1053/jhep.2000.16604

[33] Ishikawa TO, Oshima M, Herschman HR (2011) Cox-2 deletion in myeloid and endothelial cells, but not in epithelial cells, exacerbates murine colitis. Carcinogenesis 32: 417–426. 21156970 10.1093/carcin/bgq268 PMC3047239

